# The impact of immersive virtual reality meditation for depression and anxiety among inpatients with major depressive and generalized anxiety disorders

**DOI:** 10.3389/fpsyg.2024.1471269

**Published:** 2024-10-21

**Authors:** Jungjoo Lee, Junhyoung Kim, Marcia G. Ory

**Affiliations:** ^1^School of Health Professions, College of Nursing and Health Professions, The University of Southern Mississippi, Hattiesburg, MS, United States; ^2^Department of Health Behavior, School of Public Health, Texas A&M University, College Station, TX, United States; ^3^Department of Environmental and Occupational Health, School of Public Health, Texas A&M University, College Station, TX, United States

**Keywords:** virtual reality meditation, HeartMath, pilot study, emotional regulation, mental health

## Abstract

**Background:**

Mindfulness-Based Cognitive Therapy (MBCT) is a non-pharmacological approach to alleviating depression and anxiety. While technology based MBCT is a standardized cost-effective approach, there have been concerns about its feasibility and effectiveness in clinical settings.

**Aims:**

The purpose of this study was to investigate the longitudinal relationship between improved emotional regulation resulting from participation in Immersive Virtual Reality Meditation (IVRM) and Major Depressive Disorder (MDD) and Generalized Anxiety Disorder (GAD) as monitored by electrocardiogram.

**Methods:**

This study was a longitudinal single-arm clinical trial in which the intervention was conducted three times a week for 10 weeks at a behavioral health unit in a community hospital (*n* = 26). We measured Coherence Achievement Score (CAS), depression, and anxiety. The relationships between CAS, anxiety, depression, and covariates were analyzed using a Generalized Estimated Equation (GEE).

**Results:**

The findings of our study provide evidence that the CAS scores indicative of emotional regulation function after IVRM participation were associated with a reduction in depression and anxiety.

**Conclusion:**

Among the many technology-based complementary health care interventions that are available to reduce depression and anxiety, IVRM program use increases emotional regulatory function and decrease depression and anxiety.

## Introduction

Mental health conditions including depression, anxiety, and related psychiatric symptoms have become a critical global healthcare concern. The World Health Organization (WHO) estimated that 970 million adults worldwide experience mental health challenges and that mental disorders are a leading cause of disability (World Health Organization, 2022). The prevalence of depression and anxiety disorder will likely increase to over 34 million as the population ages (Center for Disease Control and Prevention, 2022). Chronic depression and anxiety symptoms are not only linked to cognitive impairment and dementia (Hayley et al., 2021; Dafsari and Jessen, 2020) but also increase the risk of cardiovascular disease (Zhang et al., 2023) and a compromised immune system (Foster et al., 2021; Eyre et al., 2016). There is a documented link between experiencing mental health challenges and healthcare utilization by older individuals receiving inpatient care and rehabilitation services who experience a 13.5% higher risk of depression and anxiety disorders (National Council on Aging, 2022).

Major Depressive Disorder (MDD) is ranked fourth among all psychiatric symptoms (McGuinness et al., 2022; Walker et al., 2015). The lifetime prevalence of MDD is over 15% and is present across the lifespan with a 20% chance of occurrence (Greenberg et al., 2015; Hasin et al., 2018). MDD is linked to dysfunction in maintaining emotional homeostasis and an increase in mortality risk of 60% (Gutiérrez-Rojas et al., 2020; Li et al., 2021; Zou et al., 2018). The impairment of the emotional regulation system caused by MCC not only triggers severe agitation, feelings of worthlessness, and recurring suicidal ideation, but also decreases average longevity (Aldao et al., 2010; Aldao et al., 2015; Berking et al., 2008; Sloan and Kring, 2007).

In addition, nearly one-half of older adults diagnosed with MDD also experience comorbid anxiety disorder (Koyuncu et al., 2019). Generalized Anxiety Disorder (GAD) is the most frequent MDD comorbidity and individuals with MDD have a 59% higher risk of GAD and a 14% higher prevalence of behavioral challenges (e.g., substance and alcohol use) (Onaemo et al., 2021; Roemer and Orsillo, 2007). GAD also weakens emotional regulation, causing comorbid behavioral challenges, and further episodes of MDD (Liu et al., 2018; Nasiri et al., 2023).

### Pharmacological and behavioral intervention approaches

There is an urgent need to design and implement effective interventions to decrease the incidence of mental health disorders in real-world settings. The most effective primary intervention for treating patients with MDD and GAD in primary care is pharmacological (e.g., antidepressant) therapy (Connolly and Thase, 2011; Dwyer and Bloch, 2019; Montano et al., 2023; Sharma et al., 2017). This approach has been found to be clinically effective in reducing the symptoms of mental health disorders of patients (Alanko et al., 2022; Leichsenring et al., 2016; Meadows et al., 2014). Despite the beneficial effects of pharmacological therapy, a growing body of literature has reported risk of harms related to the use of commonly used pharmacological treatments and has identified mild adverse side effects such as sleep disturbance, sexual dysfunction, increased blood pressure, high dropout rates, and low remission rates (Connolly and Thase, 2011; Dwyer and Bloch, 2019; Montano et al., 2023; Sharma et al., 2017). Thus, primary care patients and clinicians have stressed the importance of non-pharmacological treatments that decrease MDD episodes such as complementary and alternative medicine, exercise, or a combination of non-pharmacological treatment and antidepressant medication (Alanko et al., 2022; Leichsenring et al., 2016; Meadows et al., 2014).

Mindfulness-based cognitive therapy (MBCT) is a non-pharmacological approach to ameliorating negative emotions, GAD and MDD symptomology, and related comorbidities (Mennin et al., 2015; Saeed et al., 2019). Emotional regulation was found in systemic biomarkers as one of the neuropsychological benefits of the MBCT (Tolahunase et al., 2018). The improvements in emotional regulation realized through MBCT contribute to a reduction in depressive symptoms (Alanko et al., 2022; Leichsenring et al., 2016; Meadows et al., 2014), cortisol levels (Zhang et al., 2023), oxidative stress (Liu et al., 2015), and an increase in metabolism (Henje Blom et al., 2015), serum Brain-Derived Neurotrophic Factor (BDNF) (Autry and Monteggia, 2012) and neuroplasticity affected by MDD (Eyre and Baune, 2012).

The effect of MBCT (i.e., meditation) in preventing the relapse and exacerbation of depression has been described in longitudinal clinical trials (Meadows et al., 2014; Bédard et al., 2014; Edenfield and Saeed, 2012). Our recent pilot trial found that the MBCT was effective in reducing MDD, GDD and related mood disorders, and that the effects were maintained at a nine-month follow-up period (Renna et al., 2018). Two years of longitudinal study identified that the MBCT was effective in reducing depression and its recurrence in a follow-up assessment (Edenfield and Saeed, 2012). Moreover, MBCT reduced depression in patients with post-traumatic stress disorder (PTSD) through a three-month follow-up assessment (Bédard et al., 2014).

### Technology application

Digital health technology is a novel approach to care for mental health conditions whose feasibility and efficacy have been tested in various clinical settings (Jacobson et al., 2019; Naslund et al., 2017; Patel and Butte, 2020). Digital health encompasses a wide range of technology-based interventions (i.e., complementary treatments), precision medicine, and telehealth care that have been successfully applied to mental healthcare for individuals with MDD, GAD, and related comorbidities (Anderson et al., 2022; Arafat et al., 2021; Torous et al., 2021; Mandryk et al., 2021; Rykov et al., 2021). These technologies take on the role of predicting mental health status based on objective data over time and providing a cost-effective standardized service (Anderson et al., 2022; Arafat et al., 2021; Torous et al., 2021). Virtual reality (VR) technology is an example of a cost-effective solution for providing complementary mental health care in a consistent, standardized, precise manner (Park et al., 2019; Chirico and Gaggioli, 2019). The integration of VR technology with MCBT programs is designed to increase the efficacy of therapy for emotional regulation in MDD and GAD patients and decrease the treatment dropout rate (Colombo et al., 2021). VR interventions yield superior outcomes in managing symptoms as compared to traditional mental health care for MDD and GAD (Gorini and Riva, 2008). Recent clinical trials have provided evidence that meditation facilitated by VR technology significantly enhances depression and anxiety care (McIntyre et al., 2023; Paul et al., 2020; Paul et al., 2022). VR treatment has proven its efficacy in addressing a wide range of mental health disorders. Exposure to positive emotion in a VR meditation program was associated with a significant reduction in the agitation, depression, anxiety, and negative emotion of individuals with MDD (Chen et al., 2020; Ioannou et al., 2020).

### Gaps in current research

There have, however, been several shortfalls noted in previous studies. First, although the health benefits of CBT and MBCT as non-pharmacological treatments have been reported, little research has been conducted to investigate the benefits of immersive VR for individuals with MDD and GAD. A growing body of studies found that an IVRM program can address practical limitations to participation by: (a) accommodating individualization by allowing participants to choose from a variety of nature-based themes to navigate and interact with in over 60 different natural settings in more than 500 locations, (b) reducing physical and structural barriers, a benefit inherent to VR, by enabling participants to access nature-based settings at their preferred times and locations, and (c) addressing individual challenges associated with physical limitations (e.g., mobility, risk of falling, and pain), financial and weather-related issues, and the need for specially modified equipment (Park et al., 2019; Chirico and Gaggioli, 2019; Glegg and Levac, 2018). Even though previous literature has reported that MBCT integrated with technology is a feasible and cost-effective non-pharmacological treatment, there is, currently, a dearth of research investigating its clinical effects in patients with MDD and GAD. Second, objective measurement is required to improve the reliability and validity of psychometric assessments. The information collected from patients with MDD and GAD in previous reviews and case studies was limited due to unreliable feedback (Kandasamy and Campbell, 2022). It must also be noted that most clinical trials investigated the relationship between intervention engagement and health outcomes. However, further investigation is required to gain an understanding of the benefits of the intervention on health outcomes.

The goal of this study was to investigate the longitudinal relationship between improved emotional regulation resulting from participation in Immersive VR Meditation (IVRM) and MDD and GAD as monitored by electrocardiogram. This study will provide evidence for improved emotional regulation function related to the use of an IVRM program on the anxiety and depression of patients with MDD and GAD.

## Methods

### Samples

The sample in this study consisted of inpatients in the behavioral healthcare unit of a community hospital located in Bloomington, IN from January to April 2023. This specialized unit delivers comprehensive residential psychiatric care to individuals afflicted by a spectrum of mental health challenges including, but not limited to, schizophrenia, bipolar disorder, depression, and dissociative disorders and provides treatment for behavioral manifestations including substance abuse, contemplation of self-harm, and suicidal ideation for up to 14 days.

The inclusion criteria for participants in this study were: (a) a clinical diagnosis of MDD, and (b) confirmation of a diagnosis of GAD by the attending unit psychiatrists. Potential participants manifesting symptoms of schizophrenia, dissociative disorders, psychosis, and impairments in visual and auditory ability were omitted from the study sample.

### Study design

This study was a longitudinally designed, single-arm clinical trial in which the intervention was conducted three times a week for 10 weeks. We recruited participants based on referrals from physician progress notes and initial entrance exams. Research assistants individually contacted the patients diagnosed with MDD and/or GDD and obtained informed consent from the legal representatives of all participants before their engagement in the program. However, not every participant received the same number of interventions for two reasons: (a) even though the intervention period was 10 weeks, some participants were not able to attend every session due to their daily mental health condition and medication schedule, and (b) the physicians in charge of the emergency unit discharged some of the patient participants earlier than was planned. The discharge criteria were confidential, and the research team was not allowed to access the treatment schedule in the unit. Thus, some participants ceased participation in the intervention before the conclusion of the planned course of the intervention.

Of the initial group of 36 participants, each person, on average, engaged in around 5.1 sessions. However, 11 participants left the study without undergoing the exit assessment when they were discharged from the hospital. This left 25 participants who took part in an average of 2.7 intervention sessions which resulted in a total of 68 observations.

### Ethical consideration

This study was implemented following the ethical guidelines of the Institutional Review Board (IRB: IU#17808). We solicited and received written informed consent from the participating patients themselves or their legal representatives holding a power of attorney prior to the initiation of the data collection and treatment phases. To ensure comprehensive understanding of the study objectives and procedures by participants in the intervention group, detailed information encompassing the title, objectives, duration, type, and the nature of study participation was provided.

### Intervention

This study employed Guided Meditation VR (Cubicle, Ninjas, United States) as a technology-based innovative approach alongside traditional mindfulness programs. The Oculus Quest 2 digital headset developed by Meta was used to provide the Guided Meditation VR. The most important aspect of IVRM that distinguishes it from traditional meditation is the provision of a personalized meditation experience. Participants were able to explore natural scenery such as a green meadow, waterfall, savannah, and beach and choose a variety of nature sounds (e.g., birds chirping). Second, the IVRM provided a wide range of guided meditation programs from which participants could choose a specifically designed meditation program to release stress, assist sleep patterns, and reduce depression (Ma et al., 2023; Shaw et al., 2011). The 30-min IVRM sessions were provided according to the preference and requirements of each participant.

HeartMath (i.e., electrocardiogram) was used to measure the changes in emotional regulation related to IVRM participation. HeartMath is a biofeedback monitoring system that measures heart rhythm changes and coherence levels between sympathetic and parasympathetic activities in the autonomic nervous system (ANS) (Raghavendra and Telles, 2012). This biofeedback data allows researchers to objectively measure coherence level and related achievement scores that are associated with levels of depression and anxiety (Drageset et al., 2012; Edwards, 2016; Minen et al., 2021). The Coherence Achievement Score in HeartMath indicates changes in the level of depression and anxiety after clinical interventions.

### Measures

#### Independent variable

##### Coherence Achievement Score (CAS)

The HeartMath device measures electrical signals from the heart (i.e., electrocardiogram). HeartMath requires that a sensor be clipped to the earlobe of each participant to record electrocardiography for 3 min. The HeartMath algorithm calculates the coherence level every 5 s based on the collected data and provides an achievement score that reflects coherence for the entire session (McCraty and Tomasino, 2006; McCraty, 2022). Coherence refers to emotional regulation in which the heart and brain work together smoothly for optimal function as indicated by the harmonization between sympathetic and parasympathetic activity in the ANS (Nilsson et al., 2010). Thus, a high CAS score is indicative of a harmonized emotional state while a low score indicates an erratic and unstable emotional status (Edwards and Edwards, 2017; Edwards, 2020).

The CAS was measured both before and after each intervention, and the CAS difference was calculated by subtracting the initial from the final CAS value. Increased CAS differences indicate a boost in the consistency of the heart rhythm, implying an enhancement of the psychological and emotional balance of the participant.

#### Dependent variable

##### Patient Health Questionnaire-9 (PHQ-9)

The assessment of depression was carried out using the PHQ-9 (Lotrakul et al., 2008). The instrument is comprised of eight items, each of which is rated on a four-point scale (ranging from 0 for “not at all” to 3 for “nearly every day”). Scores on this scale vary between 0 and 27, wherein a higher score corresponds to higher levels of depression, and a score of 20 or above indicates severe depression. A diagnosis of MDD is established if the patient responds with a ‘2 = more than half the days’ or ‘3 = nearly every day’ to five or more questions. The instrument was reverse coded so that a high score indicated a low level of depression. Our research coordinator administered the PHQ-9 on two occasions, before and after each intervention.

##### General Anxiety Disorder-7 (GAD-7)

The GAD-7 questionnaire is comprised of seven items and is used to evaluate anxiety over 2 weeks (Williams, 2014). A four-point scale was employed to measure ‘Feeling nervous, anxiety, and edgy,’ ‘Trouble relaxing,’ and ‘Worrying too much’ in which a high score indicates a high level of anxiety, and a score of 15 or above denotes severe anxiety. In this study, the instrument was reverse coded so that a high score is indicative of lower anxiety levels. A trained research assistant administered the GAD-7 both at the beginning and at the end of each intervention, rather than relying on the baseline measurements provided by healthcare professionals.

### Analysis

Prior to the main analysis, we explored the demographic characteristics of study participants and conducted a descriptive analysis of the pre-post CAS gap, depression, and anxiety. The main analysis longitudinally investigated the relationship between CAS gap parameters, depression, anxiety, and covariates. The Generalized Estimated Equation (GEE) was employed to investigate the longitudinal relationship between the CAS gap (i.e., parameter), depression, and anxiety after the IVRM intervention. Age, sex, marital status, and educational level were entered as covariates into the GEE model.

The reason for using GEE is due the inability of study participants to attend every session that was provided three times a week for 10 weeks. This irregular participation caused fractional data in which the number of interventions differs between participants and were censored occasionally during the data collection. Notably, the fractional data (e.g., censored data) were inherently heteroskedastic, meaning that the variance of the outcome is tied to the values of the predictors. This results in inflated standard error estimates in both linear and logistic regression models that results in reduced statistical power for detecting a treatment effect. To address this concern (e.g., multicollinearity), applying GEE is the most appropriate approach that could be used to analyze the fractional data in longitudinal studies such as this project (Papke and Wooldridge, 2008).

The longitudinal relationships between the parameter (CAS differences), anxiety, and depression were investigated, and a 95% Confidence Interval (CI) was reported. In summary, the GEE model computed the changes in depression and anxiety as an increasing parameter (i.e., slope coefficient). All statistical analyses were conducted using SPSS version 28.0. The model equation used was:

Outcome Variable = Constant + Covariates + CAS differences

Depression, Anxiety = $\beta_{0}$ + {(Age* $\beta_{1}$ ) + (Gender* $\beta_{2}$ ) + (Marital status* $\beta_{3}$ ) + (Educational level* $\beta_{4}$ ) + (CAS differences* $\beta_{5}$ )}

## Results

### Study and intervention characteristics

Participant ages ranged from 18 to 71, with a Mean (*M*) of 42.51 and a Standard Deviation (SD) of 19.60. The sample consisted of 44.0% males (*n* = 11) and 56.0% females (*n* = 14) (Table 1). Two thirds of the participants were divorced (66.7%, *n* = 16) and on average had a high school educational level (58.4%, *n* = 14). Study participant descriptive statistics are summarized in Table 2. The average number of completed interventions per participant was 2.72 (SD = 1.20).

**Table 1:** Demographic characteristics.

Characteristics	*n*	%
Case	68	
Subject	25	
Age		
18–71 years old (Mean = 42.51, SD = 19.60)	25	
Gender		
Male	11	44.0
Female	14	56.0
Marital status		
Married	4	16.7
Separated	1	4.2
Widowed	16	66.7
Never married	3	12.5
Education		
Some high school or lower	4	16.7
High school graduate	14	58.3
Some college	5	20.8
College graduate	1	4.2

**Table 2:** Descriptive statistics.

Variables		Cohens’ *d*	Max	Min	Mean	SD
Number of intervention (i.e., index)			6	1	2.72	1.20
Independent variable						
CAS improvement (gap)			2	95	35.25	22.26
Dependent variables						
CAS	Baseline	22.25	16	166	54.69	22.95
	Exit		37	199	89.94	35.27
Depression	Baseline	6.86	21	31	26.16	2.56
	Exit		23	35	29.16	3.71
Anxiety	Baseline	4.03	8	25	20.33	4.08
	Exit		21	28	23.52	2.14

Total *n* = 25.

### Pre-post assessment differences

The CAS gap score at the exit assessment (*M* = 89.94, SD = 35.27) increased from the CAS gap at baseline (*M* = 54.69, SD = 22.95), and depression (*M* = 29.16, SD = 3.72) and anxiety (*M* = 23.52, SD = 2.14) scores increased at the exit assessment compared to baseline assessments (depression: *M* = 26.16, SD = 2.56; anxiety: *M* = 20.33, SD = 4.08). The effect size (i.e., Cohen’s *d*) is presented in Table 2. The CAS difference was 22.25 (95% CI: 1.22–1.93), with depression at 6.86 (95% CI: −1.11 to −0.23), and anxiety at 4.03 (95% CI: −1.21 – 0.31).

### Relationships between parameter estimates and depression, anxiety, and covariates

Table 3 shows the relationship between parameter estimate and depression. The unstandardized beta (*B*) represents the slope coefficient of depression increasing with CAS gap. The parameter ranged from 2 to 84 and the slope coefficient (*B*) was between −5.05 to 7.33. Depression significantly decreased with increasing CAS gap and showed a linear pattern from a parameter of 2 to a parameter of 84. The highest slope coefficient was 7.33 (SD = 0.53) at the parameter of 84 (95% CI: 129.21–394.01). The lowest *B* value was −5.05 (SD = 0.90) at the parameter of 5 (95% CI: 0.00–8.35). The B value of age was negative (*B* = −0.06, SD = 0.02, 95% CI: 0.90–0.98), indicating that older individuals had higher levels of depression. Females were found to have lower depression scores than males (*B* = 0.72, SD = 0.41, 95% CI: 0.91–4.66) and highly educated participants were found to have lower depression scores than individuals with lower education levels (*B* = 0.65, SD = 0.19, 95% CI: 1.30–2.80).

**Table 3:** Parameter estimates.

			Hypothesis test				95% Wald confidence interval for Exp (B)	
Parameter	*B*	SD	Wald Chi-Square	df	Sig.	Exp (B)	Lower	Upper
Intercept	4.48	1.06	17.85	1	0.00*	88.24	11.04	506.12
CAS gap = 84	7.33	0.53	186.88	1	0.00*	253.26	129.21	394.01
CAS gap = 58	4.13	0.70	34.33	1	0.00*	62.23	15.62	247.80
CAS gap = 54	5.25	0.37	191.36	1	0.00*	190.72	90.63	101.80
CAS gap = 51	4.62	0.43	111.51	1	0.00*	101.66	43.11	301.32
CAS gap = 38	3.18	0.82	14.86	1	0.00*	24.21	4.79	238.72
CAS gap = 36	3.16	0.47	43.51	1	0.00*	23.59	9.22	122.36
CAS gap = 35	2.18	0.84	6.62	1	0.10	8.89	1.68	60.36
CAS gap = 33	4.25	0.93	20.66	1	0.00*	70.29	11.23	46.96
CAS gap = 26	3.62	0.54	44.50	1	0.00*	37.34	12.89	439.80
CAS gap = 20	3.18	0.82	14.86	1	0.00*	24.21	4.79	108.19
CAS gap = 18	1.92	0.28	46.82	1	0.00*	6.88	3.96	122.36
CAS gap = 13	−2.18	0.35	38.69	1	0.00*	0.11	0.06	11.95
CAS gap =12	2.14	0.46	21.24	1	0.00*	8.54	3.43	0.22
CAS gap =11	−0.39	0.95	0.17	1	0.69	0.67	0.10	21.28
CAS gap =10	3.53	0.25	184.75	1	0.00*	34.17	20.54	4.39
CAS gap = 9	0.91	0.61	2.19	1	0.13	2.49	0.74	56.87
CAS gap = 5	−5.05	0.90	31.11	1	0.00*	0.01	0.00	8.35
CAS gap = 2	0							
Age	−0.06	0.02	7.79	1	0.00*	0.94	0.90	0.98
Gender	0.72	0.41	3.05	1	0.08	2.06	0.91	4.66
Marital status	0.47	0.13	11.76	1	0.00*	1.60	1.22	2.09
Education level	0.65	0.19	10.94	1	0.00*	1.92	1.30	2.80
Scale	1.52							

Dependent variable: depression. *p < 0.05.

Table 4 displays the relationship between parameter estimate and anxiety. The parameter ranged from 2 to 84, and the B value ranged from 5.60 to −0.93. The decreasing slope of anxiety was linear with increasing CAS gap scores. The highest *B* value was 5.60 (SD = 2.50) at a parameter of 84 (95% CI: 2.03–22001.12). The lowest coefficient value was −0.93 (SD = 2.88) at a parameter of 5 (95% CI: 0.08–19.82). The coefficient of age was −0.04 (SD = 0.06, 95% CI: 0.85–1.08), indicating that older participants had higher anxiety levels than their younger counterparts. The sex coefficient was −3.38 (SD = 1.66, 95% CI: 0.01–0.33), indicating that male participants had lower anxiety levels than females.

**Table 4:** Parameter estimates.

			Hypothesis test				95% Wald confidence interval for Exp (B)	
Parameter	*B*	SD	Wald Chi-Square	df	Sig.	Exp (B)	Lower	Upper
Intercept	9.87	2.65	13.79	1	0.00*	1942.63	106.08	3,5571.02
CAS gap = 84	5.60	2.50	5.03	1	0.00*	272.89	2.03	2,2001.12
CAS gap = 58	5.45	1.88	8.39	1	0.02*	232.89	5.83	9,299.42
CAS gap = 54	3.58	1.70	4.43	1	0.03*	36.02	1.28	1,010.83
CAS gap = 51	3.72	1.86	3.98	1	0.04*	41.52	1.07	1,609.87
CAS gap = 38	3.45	2.68	1.65	1	0.19	31.55	0.16	5,035.61
CAS gap = 36	4.53	1.60	8.00	1	0.00*	93.55	4.03	2,171.41
CAS gap = 35	4.57	2.92	2.44	1	0.12	96.71	0.32	2,970.44
CAS gap = 33	2.27	2.70	0.70	1	0.40	9.71	0.05	1,955.98
CAS gap = 26	3.14	1.65	3.62	1	0.04*	23.11	0.91	586.64
CAS gap = 20	2.45	2.68	0.83	1	0.36	11.61	0.06	2,220.38
CAS gap = 18	3.77	1.50	6.32	1	0.01*	43.54	2.29	826.21
CAS gap = 13	−0.34	1.51	0.05	1	0.82	0.70	0.04	13.84
CAS gap =12	2.71	1.50	3.22	1	0.07	15.01	0.78	289.13
CAS gap =11	0.82	3.02	0.07	1	0.78	2.28	0.06	860.73
CAS gap =10	0.12	1.44	0.01	1	0.94	1.12	0.06	19.17
CAS gap = 9	−0.93	1.99	0.21	1	0.64	0.39	0.08	19.82
CAS gap = 5	2.96	2.88	0.64	1	0.43	9.93	0.04	2,820.59
CAS gap = 2	0							
Age	−0.04	0.06	0.43	1	0.51	0.96	0.85	1.08
Gender	−3.38	1.16	8.48	1	0.00*	0.03	0.01	0.33
Marital status	−0.54	0.22	5.86	1	0.02*	0.58	0.37	0.90
Education level	0.23	0.44	0.27	1	0.59	1.26	0.53	2.99
Scale	11.82							

Dependent variable: anxiety. *p < 0.05.

## Discussion

This study investigated the longitudinal relationship between CAS, depression, and anxiety in MDD and GAD patients after participating in IVRM. The study results provide evidence of improvements in depression and anxiety post-intervention. Employing HeartMath as a measure of emotional regulation to measure CAS, the study also reveals that an increase in CAS, indicative of improved emotional regulation function, is associated with a reduction in depression and anxiety after IVRM participation in MDD and GAD patients.

Systematic reviews have provided evidence of the effects of non-pharmacological treatments on the emotional regulatory functions of patients with MDD and GAD (Mennin et al., 2015; Saeed et al., 2019), and prior longitudinal clinical studies found that MBCT was effective in reducing depression and anxiety in these patients (Meadows et al., 2014; Bédard et al., 2014; Edenfield and Saeed, 2012). Specifically, the use of a mindfulness activity intervention was effective in reducing depressive and anxious feelings for up to 9 months (Edenfield and Saeed, 2012). Our study results are aligned with previous research that found that the use of an IVRM program can be effective in reducing the depressive symptoms and anxiety of patients with MDD and GAD.

Prior studies have demonstrated that digital technology is an effective catalyst that can be used to enhance the effectiveness of traditional healthcare programs (Jacobson et al., 2019; Naslund et al., 2017; Patel and Butte, 2020). A growing body of literature suggests that technology based non-pharmacological treatments play an important role in improving mental health and reducing the symptoms of mental health disorders and illnesses (Park et al., 2019; Chirico and Gaggioli, 2019; Colombo et al., 2021; McIntyre et al., 2023; Paul et al., 2020). The results of our study indicate that use of an IVRM program, a MBCT in a VR environment, is associated with reduced depression and anxiety levels in patients with MDD and GAD. The application of VR technology resulted in significant effects even though the average number of interventions was low (2.72) and the discrepancy in intervention frequency was large between the patients. Thus, the findings of this study support the value of integrating VR applications into the treatment protocols of patients with MDD and GAD to achieve enhanced emotional regulation and thus, reduced levels of depressive symptoms and anxiety.

From the methodological perspective, the measurement of emotional regulation in patients with MDD and GAD has historically been challenging due to a lack of rigorous methodological strategies that produce reliable responses (Sloan and Kring, 2007; Pinho et al., 2021). This challenge has been compounded by a dearth of studies investigating the relationship between the specific benefits of MBCT applied with patients with mental health challenges. This study adopted electrocardiographic measurement (i.e., HeartMath) to objectively assess psychophysiological changes and strengthen methodological rigor. The use of HeartMath improves methodological rigor through integrating objective assessments that were lacking in previous studies (Buchanan and Reilly, 2019; Virk et al., 2018). Also, our findings conclude that CAS improvement achieved through IVRM participation leads to reduced depression and anxiety in patients with MDD and GAD. The findings of our study make a significant contribution to clinical psychology by providing empirical evidence that improved emotional regulation can be achieved using an IVRM program, and that the use of IVRM can reduce depression and anxiety in patients diagnosed with MDD and GAD.

Several limitations inherent to this study should be addressed. First, this study was a single arm clinical trial with a small sample size. While the outcomes of the study cannot be generalized and there are reliability issues, our study can serve as an initial test of the application of a technology to mindfulness therapy for MDD and GAD patients. The use of a randomized controlled trial will enable researchers to gain a deeper understanding of the health impact of IVRM, distinguishing its effects from those of traditional treatments. Second, medication that has been prescribed to patient participants by doctors may affect the changes in depression and anxiety found at the exit survey when the patients were discharged from the unit. Controlling medication during the intervention period will clarify the effect of IVRM itself. Additionally, other therapeutic activities, such as leisure education and arts and crafts activities, may have impacted our study results. Extending the pilot study to include community dwelling people with MDD and GAD will allow for comparison between inpatients and community dwellers. Third, confounders may affect the effect of IVRM program use. Technology acceptance, different stages of MDD and GAD progression, and participant demographic characteristics should be controlled or categorized. Fourth, possible validity issues arise from repeatedly using the same instrument with the same participants. Repeated use of identical instruments can sometimes lead to issues including practice effects, where participants become familiar with the test and perform better simply due to repetition, or response bias, where participants may respond consistently regardless of their true feelings or behaviors. In the future, researchers should take response bias into account when participants are being measured. Lastly, this study implemented the IVRM program without a follow-up assessment. The use of a wait listed RCT has the potential to provide deeper insight into investigations of delayed participant response over a longer time frame.

## Conclusion

Despite these limitations, we have discovered important new knowledge regarding the effects of the use of an IVRM program on the depression and anxiety of patients with MDD and GAD. Among the many technology-based complementary health care interventions that are available to reduce depression and anxiety (Anderson et al., 2022; Arafat et al., 2021; Torous et al., 2021), IVRM program use increase emotional regulatory function and decrease depression and anxiety (Park et al., 2019; Chirico and Gaggioli, 2019). The results of our study provide a rationale for implementing IVRM with patients with MDD and GAD and shed light on how mental health professionals, clinical practitioners, and caregivers can integrate VR technology into existing mental healthcare programs (Cinalioglu et al., 2023).

## Data Availability

The raw data supporting the conclusions of this article will be made available by the authors, without undue reservation.
